# Serum microRNAs are non-invasive biomarkers for the presence and progression of subarachnoid haemorrhage

**DOI:** 10.1042/BSR20160480

**Published:** 2017-02-23

**Authors:** Nian-sheng Lai, Jia-qi Zhang, Fei-yun Qin, Bin Sheng, Xing-gen Fang, Zhen-bao Li

**Affiliations:** 1 Department of Neurosurgery, The First Affiliated Hospital of Wannan Medical College, Wuhu City, Anhui 241001, China; 2 Department of Neurosurgery, Xinxiang Central Hospital , Xinxiang City, Henan 453000, China

**Keywords:** Biomarkers, MicroRNAs, qRT-PCR, Subarachnoid hemorrhage

## Abstract

miRNAs are important regulators of translation and have been associated with the pathogenesis of a number of cardiovascular diseases including stroke and may be possible prognostic biomarkers. The purpose of the present study was to determine the expression levels of miRNAs in the sera of subarachnoid haemorrhage (SAH) patients and to evaluate their relationships with the severity and clinical outcome of SAH. Serum samples on day 3 after the onset of SAH were subjected to microarray analysis with Exqion miRCURY^TM^ LNA array and quantitative PCR analysis. Serum samples from SAH patients (*n*=60) and healthy controls (*n*=10) were subjected to quantitative PCR analysis. The severities and clinical outcomes of the SAH patients were evaluated with the WFNS grade and the Modified Rankin Scale (mRS). Three miRNAs, *miR-502-5p*, *miR-1297* and *miR-4320* were significantly up-regulated in the sera of SAH patients when compared with the healthy controls. The serum *miR-502-5p* and *miR-1297* levels were significantly higher in the patients with severe SAH and a poor outcome than in those with mild SAH and a good outcome (*P*<0.05). The areas under the receiver operating characteristic (ROC) curves (AUCs) of *miR-502-5p*, *miR-1297* and *miR-4320* to distinguish the SAH patients from the healthy controls were 0.958 (*P*<0.001), 0.950 (*P*<0.001) and 0.843 (*P*<0.001) respectively. Taken together, these results indicate that *miR-502-5p* and *miR-1297* are potentially valuable indicators of the diagnosis, severity and prognosis of SAH, and *miR-4320* was a potentially valuable indicator of the diagnosis of SAH.

## Introduction

Subarachnoid haemorrhage (SAH) typically results from a ruptured aneurysm and is a small (5%) but catastrophic subset of stroke [1]. SAH is associated with 45% mortality and morbidity, occurs at a young age, and neurologic function is restored in <30% of patients. Despite advances in neurological intensive care, there is still a high prevalence of obvious neuropsychological sequelae [1]. One major determinant of the outcome after treatment of patients with SAH is the characteristics of the initial haemorrhage at admission, which significantly determines the neurological status of patients. This is an important cause of early brain injury (EBI) and early cerebral vasospasm (CVs) after SAH in humans and may be associated with delayed cerebral ischaemia (DCI) [2]. Further threats to recovery are re-bleeding, subsequent delayed vasospasm and the complexity of therapeutic intervention. Re-bleeding can be reduced by surgical or endovascular procedures and improved technology has reduced surgical complications. However, the complete pathogenesis of EBI and CVs remains incompletely understood, and no effective therapeutic method is available to adequately address these processes.

During the past few decades, the majority of experimental and clinical studies have focused on CVs, which usually occurs in 3–14 days following ictus and peaks at 6–8 days [3]. Clinical trials demonstrated that the use of an anti-vasospasm drug, such as clazosentan, was able to reduce moderate and severe cerebral angiospasm but failed to improve the clinical outcome [4]. EBI occurs within the first 72 h following the initial bleeding. The pathological derangements of EBI that contribute to an unfavourable outcome start immediately after SAH until the development of CVs [5]. Previous studies suggested that the aetiology of CVs may be linked to EBI, because there are common pathway changes and pathogenic factors in EBI and CVs [6]. This suggests the importance of pathophysiologic mechanisms in the very early phase after SAH including microvascular filling defects, the breakdown of ionic homoeostasis, inflammation and microarterial narrowing [7].

miRNAs are small non-coding RNAs that have a central role in regulating the translation and degradation of mRNAs in the cell and have significant affects on numerous physiological and pathophysiological cellular processes [8]. Changes in the expression of miRNAs in tissue or serum has been well documented in impaired smooth muscle contractility [9], endothelial inflammatory response of smooth muscle cell [10], the hippocampus after traumatic brain injury and mild cognitive impairment [11]. Recent studies have shown that the levels of certain miRNAs in the blood are associated with clinical prognosis in patients with ischaemic stroke [12,13]. Interestingly, circulating serum miRNAs may potentially be used as biomarkers to indicate tissue damage. In neuroscience, there is animal experimental data that show significant changes in miRNAs after SAH that may play a role in vascular wall changes [14]. Two miRNAs, *miR-132* and *miR-324*, were up-regulated in peripheral blood samples for both SAH DCI and SAH-non-DCI patients [15]. There was also a significant difference in the expression of some miRNAs in human cerebrospinal fluid after SAH [16,17].

Therefore, we hypothesize that SAH leads to changes in the expression of miRNAs in the brain and that these miRNAs are secreted into serum where they may serve as biomarkers for SAH. In the present study, we determined the expression profiles of serum miRNAs in patients after SAH by miRNA microarray and real-time PCR. The aim of the present study was to explore the clinical significance and prognostic value of serum miRNAs in SAH patients. The present study also identified potential novel therapeutic targets for SAH.

## Materials and methods

### Ethics approval

The present study was reviewed and approved by the Ethics Committee of Wannan Medical College, Wuhu, China and was performed in accordance with the Declaration of Helsinki. Written informed consent was obtained from all the participants.

### Study design

Adult patients (*n*=63) with confirmed SAH were included in this prospective observational study. Blood samples were collected from patients and healthy donors (*n*=13) from October 2014 to March 2015 in the Department of Neurosurgery of the First Affiliated Hospital of Wannan Medical College. Blood samples were allowed to clot at room temperature for 30 min. Serum was separated by centrifugation (5 min at 3000 ***g*** at room temperature), transferred to 1.5-ml RNase-free tubes and stored at –80°C until required for miRNA extraction. Routine management of the patients included CVs and seizure prophylaxis and early securing of the aneurysm by surgical clipping or endovascular coiling within 24 h after initiation of treatment according to the published guidelines [18].

In the initial biomarker-screening stage, miRNA microarray analysis was performed on a small set (3 compared with 3) of serum samples from SAH patients 72 h after the occurrence and normal healthy donors as the controls. Confirmation of the results was then performed using RT-qPCR. At the validation stage, samples were analysed from two groups of SAH patients, patients with World Federation of Neurologic Surgeons (WFNS) grade I–III (A, *n*=47) or WFNS grade IV–Ⅴ (B, *n*=13) (Table 1) and healthy controls (*n* =10). The patient outcomes were assessed at 9 months post-SAH using the Modified Rankin Scale (mRS). At the end of the follow-up period, the patients with an mRS score of 1–3 were classified as having a good outcome, and those with an mRS score of 4–6 were classified as having a poor outcome [19].

**Table 1:** Clinical parameters of SAH patients

Clinical parameters	Number of cases
Gender	
Male	28
Female	32
Age	
<55	20
≥55	40
Hypertension	
Yes	23
No	37
Smoking	
Yes	17
No	43

### miRNA microarray analysis

The frozen serum samples from three patients 72 h after SAH and three healthy donors were submitted to KangChen-Biotech (Shanghai, China) for miRNA microarray analysis. A previous investigation revealed that elevated miRNA expression peaked 72 h after SAH [38]. At KangChen-Biotech, total RNA was isolated using TRIzol (Invitrogen) and purified with the RNeasy Mini Kit (QIAGEN) according to the manufacturer’s instructions. RNA quality and quantity were measured with a Nanodrop spectrophotometer (ND-1000, Nanodrop Technologies) and RNA integrity was determined by gel electrophoresis. After quality control, the miRCURY™ Hy3™/Hy5™ Power labeling kit (Exiqon; Vedbæk, Denmark) was used according to the manufacturer’s guidelines for miRNA labelling. The Hy3™-labelled samples were then hybridized on to the miRCURY^TM^ LNA Array (v.18.0). The slides were scanned using the Axon GenePix 4000B microarray scanner (Axon Instruments; Foster City, CA). Scanned images were then imported into GenePix Pro 6.0 software (Axon) for grid alignment and data extraction. Replicated miRNAs were averaged and miRNAs with intensities ≥30 in all samples were chosen to calculate the normalization factor. Expressed data were normalized by median normalization. After normalization, miRNAs with different expression between the two groups were identified through fold change ≥1.5 and *P*≤0.05. Differentially expressed miRNAs between the two samples were filtered through fold change. Finally, hierarchical clustering was performed for miRNA expression profiling among the samples.

### RNA isolation

miRNAs were extracted from 400 ml of serum using the miRcute miRNA Isolation Kit (Tiangen Biotech Co., Ltd.; Beijing, China) according to the manufacturer’s protocols. An external control (CR100-01, Tiangen Biotech Co., Ltd.; Beijing, China) for miRNAs was mixed in the serum before miRNA extraction. Briefly, lysis solution (400 ml) was added to an equal volume of serum (400 ml). The aqueous phase containing total RNA was extracted with chloroform and transferred to an elution column where the protein was removed from the bound RNA. miRNAs were finally eluted in RNase-free water (30 ml), quantified with the NanoDrop 2000 spectrophotometer (NanoDrop Technologies; Houston, TX, U.S.A.) and stored at –80˚C. Only samples with an *A*_260_/*A*_280_ ratio between 1.8 and 2.0 were utilized for further analysis.

### Reverse transcription (RT)

cDNA specific for miRNA and the external control was synthesized from total RNA in a reaction mixture containing *Escherichia coli* Poly(A) Polymerase (5 units/μl) (Tiangen Biotech Co., Ltd.; Beijing, China), 5× rATP solution and 10× Poly(A) Polymerase buffer using the DNA Engine Opticon 2 Real-Time Cycler (MJ Research, Inc.; Waltham, MA, U.S.A.) with incubation at 37ºC for 60 min. Synthesized cDNA was stored at –80ºC for further analysis.

### Real-time quantitative reverse transcription-PCR (qRT-PCR)

Real-time PCR was performed using the miRcute miRNA qPCR detection kit (SYBR Green) on Applied Biosystems 7500 Real-Time PCR Systems in triplicate for each sample, using 0.5 μl either a forward primer specific to the miRNA or the external control, 10 μl 2× miRcute miRNA Premix (with SYBR and ROX), 0.4 μl reverse primer (10 μM), cDNA and water to a final volume of 20 μl (all from Tiangen Biotech Co., Ltd.; Beijing, China). The reactions were set up in 96-well plates and incubated at 94ºC for 2 min, followed by 40 cycles of 94ºC for 20 s and 60ºC for 34 s. Cycle threshold (*C*_t_) values were determined, and the expression levels were calculated in triplicate from the equation 2^–Δ*C*^_t_, where the raw data of the target miRNA were normalized to the *C*_t_ of the external control [20]. PCR reactions were separated by agarose gels to determine the product size, and dissociation curves were used to examine the specificity of the qPCR assay.

### Statistical analysis

All data were analysed using MedCalc version 13.0.0 (Broekstraat 52, 9030; Mariakerke, Belgium). Data were presented as mean ± S.D. Differences between the two groups were assessed by the Mann–Whitney U test, and multiple comparisons among more than two groups were performed using the Kruskal–Wallis test. The correlations among the variables were calculated using Spearman correlation coefficient analysis. The receiver operating characteristic (ROC) curves were generated for the combined miRNAs and the clinical parameters (age, smoking, gender and hypertension), which were calculated with a comprehensive probability using multiple regression among cases and controls (Table 1). The areas under the ROC curves (AUCs) were calculated to evaluate the predictive powers of the candidate miRNAs for SAH. The data were regarded as statistically significant at a value of *P*<0.05.

## Results

### miRNAs expression in serum from SAH patients in the screening phase

To select candidate miRNA biomarkers for SAH patients, we first performed initial genome-wide miRNA screening of two pools of serum samples derived from SAH patients and healthy controls by miRNA microarray analysis. The expression of 2086 miRNA genes were analysed by miRNA microarray. Scanned images were then imported into GenePix Pro 6.0 software (Axon) for grid alignment and data extraction. Significant differentially expressed miRNAs between the two groups were identified as those with a fold change ≥1.5 and *P*≤0.05. Using these criteria, we identified 13 miRNAs that were differentially expressed among the different groups and all of these differentially expressed miRNAs were up-regulated (Table 2).

**Table 2:** miRNAs differentially expressed in the two groups

ID	Name	Fold change (SAH compared with control)	*P* value
11134	*hsa*-*miR-502-5p*	2.23	0.02095611
147705	*hsa*-*miR-4320*	4.91	0.027443852
148206	*hsa*-*miR-3664-5p*	2.04	0.002071489
168670	*hsa*-*miR-4694-5p*	1.68	0.048549787
169132	*hsa*-*miR-382-3p*	2.47	0.016512086
46944	*hsa*-*miR-1297*	4.99	0.033294243
168644	*hsa*-*miR-4775*	1.90	0.032430597
169247	*hsa*-*miR-4477a*	5.01	0.036226663
169048	*hsa*-*miR-4736*	1.78	0.011571575
147809	*hsa*-*miR-514b-3p*	1.53	0.021561591
10916	*hsa*-*miR-1-3p*	2.63	0.025191029
148349	*hsa*-*miR-3938*	1.67	0.009873719
168850	*hsa*-*miR-3191-5p*	2.95	0.01330053

### Validation of microarray data by qRT-PCR

**Figure 1:**
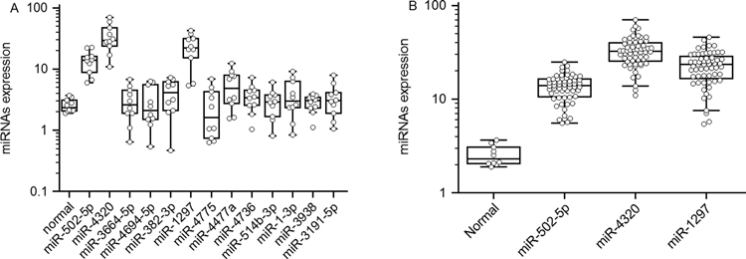
Expression profiles of the serum miRNAs after SAH (**A**) The results of the miRNA microarray were validated using serum samples from a cohort of patients and controls. (**B**) The differences in the concentrations of *miR-502-5p*, *miR-1297* and *miR-4320* for SAH patients and healthy controls were validated using serum samples from a larger cohort of patients and controls.

As described above, miRNAs have been suggested as potential disease biomarkers because they can be detected from the serum. To evaluate the miRNA microarray results, expression of miRNAs from a total of 20 serum samples were analysed, including samples from patients with SAH (*n*=10) and samples from normal control subjects (*n*=10). We analysed whether these candidate miRNAs could serve as circulating markers by comparing their serum levels between SAH patients and normal controls. qRT-PCR confirmed that the three miRNAs (*miR-502-5p*, *miR-1297* and *miR-4320*) showed statistically significant changes in expression between the experimental group (72 h post-SAH) and healthy controls (Figure 1A). The other miRNAs showed no significant differences in expression between the control and the serum samples of 72 h post-SAH.

Subsequent technical confirmation of the data with additional qPCR assays with a larger group of samples (60 samples post-SAH and 10 samples of normal controls) confirmed that *miR-502-5p*, *miR-1297* and *miR-4320* exhibited significantly altered expression levels after SAH when compared with controls (Figure 1B).

### Relationships of *miR-502-5p* and *miR-1297* with SAH severity

**Figure 2:**
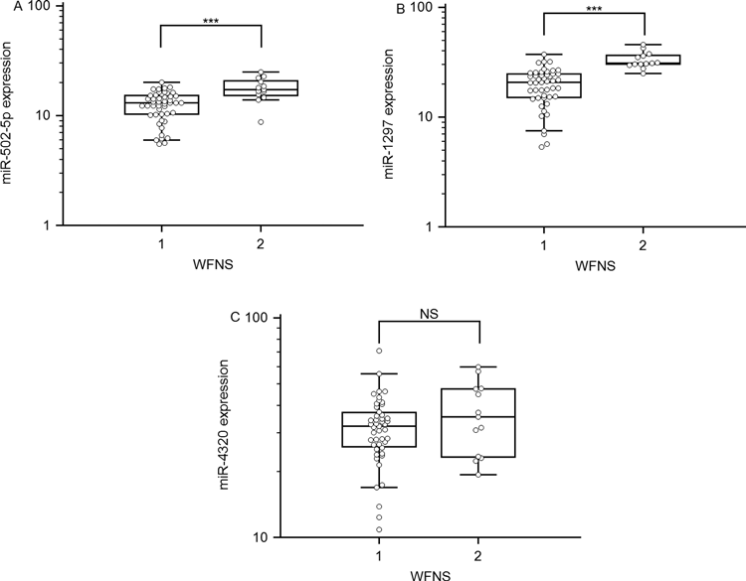
Relative levels of *miR-502-5p*, *miR-1297* and *miR-4320* in patients with severe SAH (high WFNS grade) and those with mild SAH (low WFNS grade) (**A**–**C**) Serum samples were collected 72 h after SAH and the miRNAs were assayed by qRT-PCR; ***P<0.001; NS, no significance.

The WFNS grade is a tool that is used to assess the level of brain injury after SAH and higher scores denote greater impairment. Because understanding severity and progression are important for the future treatment of SAH patients, the levels of these three miRNAs were analysed with respect to different groups classified according to disease severity. No significant association between *miR-4320* expression and SAH severity was identified. However, as illustrated in Figure 2, comparison of the SAH patients with the controls revealed that the serum levels of *miR-502-5p* and *miR-1297* were all significantly increased (*P*<0.001). To further investigate the relationships between the expression levels of these two miRNAs and WFNS grade, Spearman correlation coefficient analysis was performed. The results revealed that in the SAH patients, the serum level of *miR-502-5p* (ρ =0.60; *P*<0.001; 95% CI: 0.41–0.74) and *miR-1297* (ρ =0.75; *P*<0.001; 95% CI: 0.61–0.84) were negatively correlated with SAH severity as scored by WFNS grade.

### Relationships of *miR-502-5p* and *miR-1297* with clinical outcomes of SAH patients

**Figure 3:**
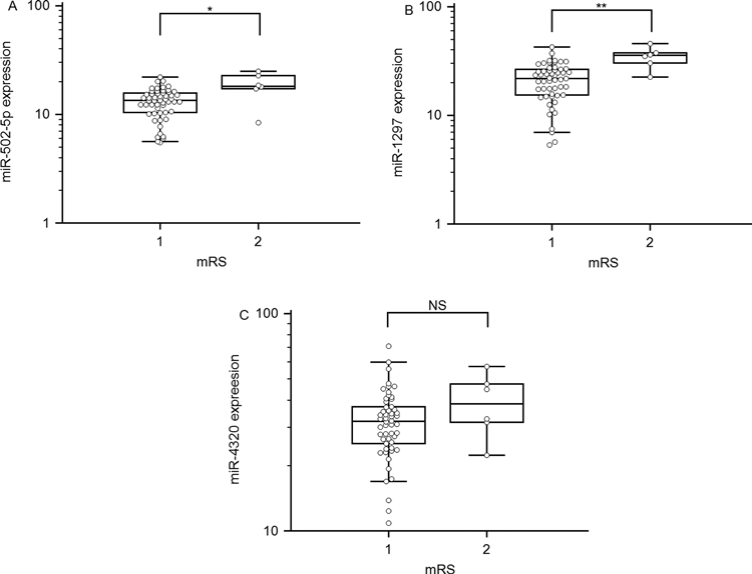
miRNAs serum levels in relation to patients’ clinical outcomes The changes of miRNAs were analysed by qRT-PCR. Data are presented as the mean ± S.D.; *P<0.05, **P<0.01 or NS, no significance.

It is important to determine the potential outcomes of SAH patients at the earliest possible stage to optimize the treatment. Thus, the patients were divided into two groups according to their clinical outcomes. As shown in Figure 3,* miR-502-5p* and *miR-1297* levels were directly associated with poor outcome (both *P*<0.001). The mRS scores were lower in the patients with lower expression of *miR-502-5p* and *miR-1297* than in those with higher expression. Therefore, outcome prediction may be improved by determination of the levels of these serum miRNAs.

### ROC analysis of serum *miR-502-5p*, *miR-1297* and *miR-4320* levels in SAH

**Figure 4:**
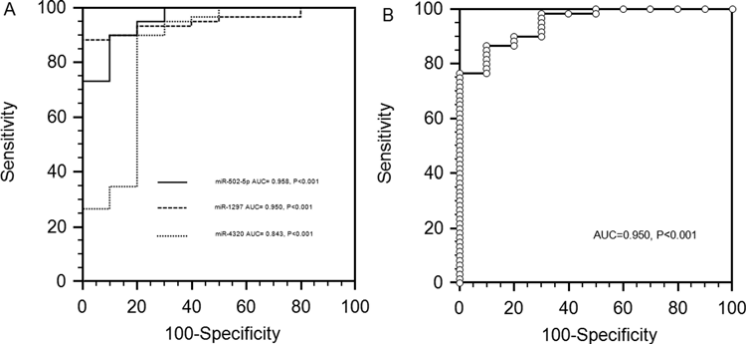
ROC curves to distinguish SAH patients from healthy controls

The diagnostic potential and discriminatory accuracy of serum *miR-502-5p*,* miR-1297* and *miR-4320* were evaluated by ROC curve analysis and the corresponding AUC values. ROC analyses revealed that serum *miR-502-5p*, *miR-1297* and *miR-4320* levels were robust in discriminating patients with SAH from healthy controls, with AUC value of 0.958 (95% CI: 0.882–0.992; *P*<0.001), 0.950 (95% CI: 0.870–0.988; *P*<0.001) and 0.843 (95% CI: 0.737–0.919; *P*<0.001) respectively (Figure 4A). Additionally, ROC curves revealed that the combined expression of these miRNAs and the clinical parameters were robust in discriminating patients with SAH from healthy controls, with an AUC value of 0.950 (95% CI: 0.870–0.988; *P*<0.001) (Table 1, Figure 4B).

## Discussion

miRNAs are reported to act as regulators of protein-coding gene expression and have been shown to affect several important biological processes that are associated with traumatic brain injury-induced pathophysiology, including apoptosis and neuronal plasticity [21]. DNA or protein profiling of SAH patients have not successfully elucidated the pathogenesis of SAH. Until now, there are only few reports analysing changes in miRNA for SAH patients. In the present study, we aimed to analyse the miRNA expression profiles in the peripheral blood of the patients after SAH and people without SAH to develop a further understanding of the molecular mechanisms involved in SAH.

The present study analysed miRNA expression profiles in the peripheral blood of the patients after SAH and normal control subjects using miRNA microarray technology to identify changes in the expression of miRNAs. We identified 13 miRNAs that were differentially expressed among the different groups and these 13 miRNAs were all up-regulated. These microarray results for *miR-502-5p*, *miR-1297* and *miR-4320* were verified by qRT-PCR experiments. Our results revealed that the serum levels of *miR-502-5p* and *miR-1297* were markedly increased in the SAH patients compared with the controls in association with severity and clinical outcome. Additionally, these miRNAs could discriminate the SAH patients from the healthy subjects. To our knowledge, this is the first study of the relationships among the levels of serum miRNAs and the clinical outcomes of SAH patients. Our results suggest that *miR-502-5p*, *miR-1297* and *miR-4320* may serve as novel biomarkers for diagnosis and progression of SAH and these miRNAs may be involved in SAH pathogenesis.

We also found that the serum levels of *miR-502-5p* and *miR-1297* in SAH patients with higher WFNS grade were higher than those with lower WFNS grade. Moreover, the serum levels of these two miRNAs were higher in patients with a poor clinical outcome than in those with a good outcome. These results suggest that these miRNAs, especially *miR-502-5p*, may participate in the occurrence, development and repair progression of SAH. The pathogenesis of SAH is complex and involves inflammation, apoptosis, altered plasticity and neuronal regeneration. *miR-502-5p* has been reported to be involved in many physiological processes via the modulation of cell-cycle progression, cell proliferation and signal transduction [22]. Originally, *miR-502* was reported to be a tumour-related miRNA that inhibited several tumour-associated proteins, such as histone methyltransferase SET8, TP53 codon 72 polymorphism, tumour necrosis factor receptor associated factor 2 (TRAF2) and was involved in many processes, including apoptosis, proliferation, motility and invasiveness [23–25]. Additionally, one study demonstrated that *miR-502-5p* inhibited the NF-κB signalling pathway and may prevent IL-1b-induced chondrocyte injury by targeting TRAF2[23]. The overexpression of *miR-502-5p* caused anti-apoptotic, anti-catabolic and anti-inflammatory effects in IL-1b-induced chondrocytes. *miR-1297* is deregulated in a variety of tumour tissues. This miRNA may influence cellular function through diverse pathways, as several targets of *miR-1297* have been reported including the phosphatase and tensin homologue deleted on chromosome ten (PTEN) [26], cyclo-oxygenase-2 (Cox-2) [27], high mobility group protein A1 (HMGA1) [28], HMGA2 [29], astrocyte elevated gene-1 (*AEG-1*) [30], xCT [31] and histone-lysine *N*-methyltransferase (EZH2) [32]. An involvement of *miR-1297* has been reported in the deregulation of the glioma that may prevent proliferation and invasiveness by targeting HMGA1 [28]. A newly described *miR-1297*/*AEG-1*/Wnt signalling pathway has been described with a role in prostate cancer [30]. Finally, *miR-1297* modulates the redox regulation of stem-like cells through targeting xCT in the CD44v-xCT axis in colorectal cancer [31].

There are multiple theories associated with the development of the severity and prognosis in SAH. Each represents a possible source of reliable biomarkers, including endothelin, matrix metalloproteinase-9 and protein S100 [33–35]. However, it is unlikely that a single biomarker is realistic. miRNAs are released in response to biochemical processes and can be detected in serum. miRNAs may play a direct role in the regulation of systemic biological processes such as those related to SAH. A role for different miRNAs may connect the disparate theories regarding the pathological derangements of SAH. Indeed, studies have suggested a role for miRNAs in inflammation and apoptosis [36,37]; both processes have been linked with the progression of SAH. Therefore, further investigation of the role of miRNAs in pathogenesis and the development of SAH is warranted.

The findings from the present study may be limited for several reasons. First, the present study included only a small number of Chinese patients, so the sample size is moderate. Second, the samples were collected from patients after surgery and during drug therapy. The surgical treatment and drugs may induce changes in the expression levels of miRNAs. Third, an examination of changes in the levels of these miRNAs over a longer time period will help to determine the relationships among these miRNAs and the progression of this condition. In the present study, although the clinical outcomes of the SAH patients were assessed at 9 months post-SAH using the mRS, the miRNA levels were measured 72 h post-SAH. Finally, future studies should assess the relationships of the expression levels of these three miRNAs relative to measures of severity based on assessments other than the WFNS, such as the duration of coma and the duration of post-traumatic amnesia.

In summary, the present study showed differences in miRNAs expression in serum in response to SAH. These findings suggest that miRNAs may play important roles in SAH. Further studies on the miRNAs identified herein may enhance our understanding of the miRNA-based mechanisms of SAH and may provide candidate targets for future clinical applications.

## Abbreviations

AEG-1: astrocyte elevated gene-1
AUC: areas under the ROC curves
CD 44v: Cluster of differentation 44 variant
*C*_t_: cycle threshold
CVs: cerebral vasospasm
DCI: delayed cerebral ischaemia
EBI: early brain injury
HMGA1: high mobility group protein A1
HMGA2: high mobility group protein A2
hsa: Homo sapien
IL-1b: interleukin-1b
miR: microRNA
mRS: Modified Rankin Scale
qRT-PCR: quantitative reverse transcription PCR
ROC: receiver operating characteristic
ROX: ROX reference dye
RT-qPCR: real-time quantitative polymerase chain reaction
SAH: subarachnoid haemorrhage
SET8: histone methyltransferase SET8
S100: soluble-protein 100
TP53: Tumor protein 53
TRAF2: tumour necrosis factor receptor associated factor 2
WFNS: World Federation of Neurologic Surgeons
xCT: interface-localized charge transfer protein
95% CI: 95% confidence interval
